# Intervention and evaluation of the life design counseling: A case study

**DOI:** 10.3389/fpsyg.2022.1045898

**Published:** 2023-01-04

**Authors:** Danqi Wang, Xiping Liu

**Affiliations:** Faculty of Psychology, Tianjin Normal University, Tianjin, China

**Keywords:** life design counseling, career adaptability, career construction interview, Innovative Moments Coding System, Future Career Autobiography

## Abstract

This article aims to explore the effectiveness of Life Design Counseling (LDC) for a high school student before choosing the subject. To evaluate LDC outcomes, the Career Adapt-Abilities Scale-China Form was used before and after the intervention. Two measures were used to evaluate the process of LDC: the Innovative Moments Coding System (IMCS) and Future Career Autobiography (FCA). The results show that the LDC approach produced a significant change in career adaptability. In addition, the findings demonstrate a significant narrative movement or change with the evaluation of the process. Implications for future research and practice are discussed.

## 1. Introduction

Beginning in the 20th century, career guidance was centered on the “person-job matching” model, which meant making rational decisions based on self and career information. Since the middle of the 20th century, career counseling has been based on career development theory, which emphasizes that career is a continuous and evolving process. In the 21st century, career intervention is centered on the career construction theory of life design counseling, which focuses on personality traits, career adaptability, and life themes.

## 2. Literature review

### 2.1. Life design counseling

Life design counseling (LDC) combines the theory of career construction and self-construction that describe career development (Guichard, 2005; Savickas, 2005). Career construction theory asserts that the individual career construction process corresponds to three levels of the psychological self: social actor, motivated agent, and autobiographical author (Savickas, 2013). When an individual's career is hindered, he needs to reflect on the past and make adjustments to rewrite his career. At this time, an individual can be regarded as an autobiographical author, who can reconstruct the future by deconstructing the past and finding the direction of career development (Yang et al., 2021).

The five presuppositions of LDC are contextual possibilities, dynamic processes, non-linear progression, multiple realities, and personal patterns. In addition, the framework of the LDC is life-long, holistic, contextual, and preventive (Savickas et al., 2009). The intervention model for life design relies on stories and has six steps. First, the client and counselor need to define the problem and desired goals. In the process, they establish a counseling relationship as a working alliance and listen to the client's career story to help his/her reflect on the theme and meaning of the story. In the second step, the client is encouraged to reflect on experiences and expectations, actions and interactions, relationships with others, and expectations for the future to help the client shape his/her career story. The third step aims to open new perspectives and allow the client to rewrite the story by interpreting it from a different perspective. The fourth step is to place career dilemmas encountered in the rewritten story. The problem may be solved when the issue comes into a new view. The fifth step is to craft a specific plan, which should include how to deal with current obstacles and a narrative for rehearsing a new life story. The sixth step consists of follow-up, both short-term and long-term (Savickas et al., 2009). The life design intervention constructs a career through small stories, reconstructs the stories into a life portrait, and co-constructs intentions that move the career story into a new episode (Savickas, 2012). Savickas (2013) categorized the process of life design counseling as constructing, deconstructing, reconstructing, co-constructing, and action.

LDC aims to help clients explore possible careers, reshape their narrative through meaning construction, clarify their self-concept, establish their life purpose, and create a meaningful life (Savickas, 2012). LDC is divided into three categories: process intervention, process evaluation, and results evaluation (Wen et al., 2022).

### 2.2. Process intervention

The process intervention on LDC mainly includes Career Construction Interview (CCI). There are five main topics of CCI: (1) role models; (2) favorite magazines, television shows, or websites; (3) favorite story from a book or movie; (4) mottos; and (5) early recollections from 3 to 6 years old (Savickas, 2011).

### 2.3. The result of evaluation

From the aspect of the quantitative evaluation, the result of the evaluation is to compare the clients' status after and before the counseling to find out the differences. Maree (2015) coded four college students' career stories for thematic analysis, and the results demonstrated that career construct counseling was able to bring about change in the participants' career stories. From the aspect of the LDC evaluation, researchers found that the clients' career choices indecision (Maree, 2020), anxiety, uncertainty, and insecurity decreased (Obi, 2015; Barclay and Stoltz, 2016), while sense of self (Maree, 2016; Maree and Twigge, 2016; Maree et al., 2018), career adaptability increased (Maree and Crous, 2012; Maree and Gerryts, 2014; Maree, 2021). However, there are some discrepancies between the results of different studies. The study showed that LDC did not produce a significant change in career adaptability (Cardoso et al., 2018).

Career adaptability is described as “a psychosocial construct that denotes an individual's resources for coping with current and imminent vocational development tasks, transitions, traumas” (Savickas and Porfeli, 2012). Career adaptability has four sub-dimensions: career concern, career control, career curiosity, and career confidence. The four sub-dimensions correspond to the four career questions: “Do I have a future,” “Who owns my future,” “What do I want to do in the future,” and “Can I do it.” Career concern essentially refers to career future and relates to the belief that their future is worth preparing for. Career control indicates when individuals believe they are responsible for constructing their careers. Career curiosity involves exploring career options, exploring opportunities to realize these opportunities, and active engagement with these choices. Career confidence refers to self-efficacy concerning one's ability to construct their future and overcome possible difficulties (Savickas, 2013).

### 2.4. The process of evaluation

The process evaluation of life design counseling used IMCS to analyze the change in order to assess the effectiveness of the counseling. The researchers developed the Innovative Moments Coding System (IMCS) to track IMs throughout the treatment. Individuals who begin to think differently and explore new ways of behaving and connecting would be considered innovative moments (IMs). Five categories of IMs were previously identified, including Action IMs, Reflection IMs, Protest IMs, Reconceptualization IMs, and Performing Change (Gonçalves et al., 2011). The IMCS was used to conduct an intensive analysis of the narrative shift in a case of life design counseling (Cardoso et al., 2014b). Studies using the IMCS have shown that the coding system has good reliability across therapeutic and diagnostic and proved to be a reliable method for studying short-term psychotherapeutic change processes (Cardoso et al., 2014a).

Future Career Autobiography (FCA) is another method of evaluating processes, as it is an occupational narrative tool designed to measure and identify how occupation narratives change over time. The word count analysis of Rehfuss and Di Fabio (2012) supports the validity of FCA.

Meta-analysis showed that individual counseling was more effective than other modalities (Whiston et al., 1998, 2017). There is little research on individual career counseling in China. Chen (2020) provided individual career counseling to a high school student, but the counseling lacked the evaluation of the intervention process and outcomes.

Therefore, the present case study implemented life design counseling (construct, deconstruct, reconstruct, co-construct, and action) for a career intervention approach with a high school student and tested its effectiveness through outcome evaluation and process evaluation.

## 3. Methods

### 3.1. Participant

The client was a 16-year-old student who had just entered high school in her first year. During the first semester, she must declare to select one subject from physics and history and then choose two subjects from chemistry, biology, politics, and geography. Selecting subjects determines the choice of major in the future.

Her father urged her to select physics for employment. Sometimes she believed history might be a better choice, but physics seemed more advantageous for employment. She was attracted by writing and wanted to become a writer in the future. Likewise, she was also interested in law and hoped to become a lawyer in the future. In this way, she must choose history as a writer or lawyer. She needed help to make a choice and wondered whether history would be the best option for her. The main characteristics of the client are scattered interests and indecisiveness in choosing subjects.

### 3.2. Measures

#### 3.2.1. Results' evaluation: Career Adapt-Abilities Scale-China Form

Career Adapt-Abilities Scale-China Form (CAAS-CF; Hou et al., 2012) measures the result evaluation of life design counseling. The Chinese version of the CAAS contains 24 items: six items of career concern, career control, career curiosity, and career confidence, respectively. In addition, it is a five-point Likert scale in which the higher scores indicated more career adaptability. Thus, the alpha coefficient of the total scale was 0.89. The four sub-scales registered internal consistencies of 0.64–0.79.

#### 3.2.2. Process evaluation: Innovative Moments Coding System

The CCIO was designed to evaluate the outcomes of life design counseling intervention (Di Fabio, 2016). It includes seven questions. “What modalities or content do you think could be helpful to you? What are your main useful resources? What are the main obstacles you encounter? Who do you think has been helpful to you? What do you think would be helpful to you? What do you think are the main challenges you face? What are the main goals you hope you can achieve?” The CCIO and IMCS-based coding systems indicate that the assessment is a practical qualitative approach (Di Fabio, 2016).

The categories and its definition are as seen in Table 1.

**Table 1:** Definition of innovative moments (Cardoso et al., 2019).

**IM level**	**Subtype**	**Definition**
Level 1 (creating distance from the problem)	Action 1	Behaviors to overcome the problem
	Reflection 1	New understandings of the problem
	Protest 1	Object to the problem
Level 2 (centered on the change)	Action 2	Generalization into the future and actions of good outcomes
	Reflection 2	Contrasting the self and transformation process
	Protest 2	Assertiveness and empowerment
Level 3 (consolidating change)	Reconceptualization	Reposition outside the problematic experience and understand the transformation

#### 3.2.3. Process evaluation: Future Career Autobiography

Future Career Autobiography is a qualitative instrument (Rehfuss, 2009). It has only one question “What do I think about my future?......” FCA is a narrative tool designed to identify and measure changes in an individual's career narrative, providing a quick and easy narrative method to assess the effectiveness of career interventions. FCA consists of Eight Degrees of Change themes: General Fields and Desires to Specification and Exploration, General Interests to More Specification, Non-descript “Job” to Specification, Disregard to Direction, Vagueness to Focus, Hindered to Hopeful, Fixation to Openness, and Stagnation.

## 4. Procedure

Life design intervention was held weekly for about 50 min of three sessions. Before the formal counseling, the goals and informed consent were mainly explained to the client, and then she completed the Career Adapt-Abilities Scale-China Form (CAAS-CF), Career Counseling Innovative Outcomes (CCIO), and Future Career Autobiography (FCA). After the counseling, the client completed the CAAS-CF, CCIO, and FCA.

The intervention was based on the strategy described by Savickas (2013) in a structured interview. The planned sequential intervention objectives and questions for the interview can be seen in Table 2 below.

**Table 2:** Life design counseling plan.

**Stage**	**Intervention**	**Questions for the interview**
Session 1	Constructing and deconstructing	Q1. Who were your three role models, or whom did you admire? What are their traits?
		Q2. What is your favorite (a) magazine, (b) TV program, and (c) app? Why?
		Q3. What is your favorite story in book or movie? Why?
		Q4. What is your favorite motto or quotation? Adapt this motto in your own words.
		Q5. What are your earliest recollections? I would like to hear you tell a story that happened before you were 6 years old and then add a title to the story.
Session 2	Reconstructing	Q1. Thinking about the problems you face, what does your deepest concern?
		Q2. Describe yourself with three adjectives. How can you change your traits in order to solve the problem?
		Q3. What kind of person do you like to be with? What kind of place do you like to stay?
		Q4. What are the three careers you are most interested in?
		Q5. If you adopt the script of your favorite story, so what will happen to you?
		Q6. In connection with your favorite motto, what is the best advice you give yourself right now?
Session 3	Co-constructing and action	Q1. What traits do you have?
		Q2. Where do you want to work?
		Q3. What do you want to do there?
		Q4. Why do you want to do the work?
		Q5. How should you start?
		Q6. What are your goals?
		Q7. What strategies do you intend to accomplish the goals?
		Q8. What difficulties might you encounter? How do you solve them?
		Q9. What is the time schedule?
		Q10. What are the available resources for you?

## 5. Ethical issues

Written informed consent from the client's parents and permission from the client were obtained for the analysis of the data and the reporting of the findings, which was approved by the Governing Body and the Principal of the school that the participants attend, and permission was granted for the anonymous publication of the findings.

## 6. Results

### 6.1. Process evaluation: Innovation Moment Coding System

The process evaluation of IMCS includes seven questions, such as “What modalities or content do you think could be helpful to you?” The Pre-test and Post-test of the IMCS are mainly shown in Table 3.

**Table 3:** The pre-test and post-test of the Innovation Moment Coding System.

**Questions**	**Pre-test**	**Post-test**	**Coding results**
1. What modalities or content do you think could be helpful to you?	Communication	Identify and solve weak subjects	Reflection 2 IMs
2. What are your main useful resources?	Phones, books	Parents	Reflection 2 IMs
3. What are the main obstacles you encounter?	I have expectations for the future, but at the same time, I am confused. I want to grow up to do more and realize some of my ideals, but I am afraid that the future will be difficult, I will be in a dilemma, I will not reach their goals, and I will lose hope in life.	Mathematics subject	Reconceptualization IMs
4. Who do you think has been helpful to you?	Teachers	Myself	Protest 2 IMs
5. What do you think would be helpful to you?	Books	The plan of action	Reflection 2 IMs
6. What do you think are the main challenges you face?	College entrance exams, stress, confusion	Mathematics subject	Reconceptualization IMs
7. What are the main goals you hope you can achieve?	Improve my writing skills and have a successful entrance exam	Rising performance in mathematics and top 100 overall grade level.	Reflection 2 IMs

### 6.2. Results' evaluation: Career adaptability

Career adaptability was measured using Chen (2021) norm as a reference. Brown (2015) proposed Zcs values for assessing the clinical impact of intervention cases. The client's career concern changed from below 1.66 standard deviations to 0.21 standard deviations, career control changed from below 1.28 standard deviations to below 0.51 standard deviations, career curiosity changed from below 1.16 standard deviations to below 0.2 standard deviations, career confidence changed from below 1.41 standard deviations to below 0.68 standard deviations, and career adaptability changed from below 1.58 standard deviations to below 0.33 standard deviations. The change in values indicates that life design counseling can improve the client's career adaptability. In general, the pre-test and post-test changes in career adaptability can be shown in Table 4.

**Table 4:** Pre-test and post-test changes in career adaptability.

	**Rank**	**Pre-test**	**Post-test**	**Z_cs_Pre**	**Z_cs_Post**	***M***	**SD**
Career concern	1–5	2.67	4	−1.66	0.21	3.85	0.71
Career control	1–5	3.17	3.67	−1.28	−0.51	4.00	0.65
Career curiosity	1–5	3	3.67	−1.16	−0.2	3.81	0.70
Career confidence	1–5	2.83	3.33	−1.41	−0.68	3.79	0.68
Career adaptability	1–5	2.92	3.67	−1.58	−0.33	3.87	0.60

### 6.3. Process evaluation: Narrative changes

When asked about “What I think about my future is......” Before the counseling, the client wrote, “I want to be a famous writer or a musician, and my parents are well”. After the counseling, she wrote, “After my job is stable, I will buy a car and a house mainly by my efforts, and after I settle down with my parents, I will write a book when I am 27 and start a family when I am 30 years old”.

The results indicated a significant narrative change in the post-FCA, moving from general to more specific occupational themes, vagueness to focus, and fixation to openness. The pre-FCA word count is 11, post-FCA word count is 45. Word count analysis revealed expanded narrative expression.

## 7. Discussion

The present study examined the effectiveness of LDC for high school students who were experiencing subject choice indecision. From the findings of this study, we concluded that high school students who experience subject choice indecision could benefit from LDC to reconstruct their career stories with meaningful career goals.

In the first counseling of CCI, The client stated that her father had toughness for hard work. Her classmate is also a role model who is kind and good at studying. In addition, Xuanyi Wu, in the 101 Girls' Team, is very hardworking and has team spirit with others. The counselor asked the client about her favorite books, Apps, and television shows to assess her preferred environments and interests. She liked to read some light serialized novels on the Zhihu App. Her favorite television show was Running Man because the participants are united in accomplishing tasks for a common goal. When asked about her favorite story, she said it was The Soulmate, the story of a girl who dedicates a lot to her friend. Her motto is that success is equal to 99% hard work plus one percent talent. The client reported the following early recollection, “When I graduated from kindergarten, my teacher gave me a set of encyclopedias, which touched me so much that I enjoyed reading and writing”.

The role model trait in the first counseling represented self-ideal. When asked about role models, the client blurted out, “my father.” The characteristics of her father are perseverance and hard work, which are the ideal traits of the client. The most preferred App is close to the actual life of the client and reveals the preferred work environment that suits the individual's style. The answers indicate that the client prefers text-related work in a relaxed and cohesive environment. The two questions provided insight into the client's self-concept and work environment, while their favorite movie was how to play the self in the atmosphere by executing the script, a connection between the self and the environment. The movies that the client likes are all scripted stories about being righteous to friends, which is the way the client aspires to handle things. The motto created by the client was that success requires four factors: self-confidence, hard work, perseverance, and interest, which will be the strategy and action guide for the client to achieve the goal. The early memory of the client is that her kindergarten teacher gave her a set of encyclopedias when she was 6 years old and named it “the gift of childhood,” which shows the importance of the gift. It explains why the client likes reading and writing and is also the main reason why she repeatedly struggles and perseveres when she encounters learning difficulties.

The case comprehended her interest in her favorite App, and her career concern was improved. The motto made the case realize that she is the one who leads her career development, so the case's career control was improved. In addition, she saw possible scripts for problem-solving and gained career confidence in the early stories. Through career counseling, she became more willing to actively explore herself and the world of work, which increased the individual's career curiosity. Therefore, the career adaptability of the case was increased.

In the second counseling of reconstruction, the client integrated reflections on the past, present, and future, forming a life portrait and reconstructing a new career story. In the life portrait, the client recognized that the low grade was due to avoidance of studying math and being afraid to ask the teacher questions, which is a lack of career confidence. The occupational ranking of the cases was writer, lawyer, and civil servant, but the work environment the client expected was hilarious, which obviously did not match the occupational expectations. The client was then prompted to consider whether there is a work environment that is lively and, at the same time, satisfies the hobby of writing.

In the third session, the life portrait was re-narrated with the client, and the reconstructed self-identity was depicted through the portrait. It formed the continuity of the career narrative while the client recognized the possibilities for career development. The continuity of the career narrative is created while the case sees the possibility of career development. After discussing with the client about the possibility and methods to improve her grades, she realized that her career problem would be solved if her grades in mathematics improved.

The findings show that life design counseling by constructing, deconstructing, reconstructing, co-constructing, and action is especially relevant for high school students who are facing career transitions. More particularly, life design interventions can increase individual sources such as career adaptability (Rossier, 2015). The findings support Maree and Twigge (2016) finding that life design counseling promotes self-awareness. Maree et al. (2018) highlighted the value of life design counseling in developing a clearer sense of self. Likewise, Maree and Gerryts (2014) demonstrated the value of the LDC in enhancing the career adaptability of clients. Similarly, the findings in the present study support the findings of Maree (2021), who confirmed the critical role of life design counseling in improving students' career adaptability. The current findings are also in line with the findings of Cardoso et al. (2014a) that IMCS is suitable for analyzing client changes during career interventions. Lastly, the findings reported in this article indicate that the Future Career Autobiography of the qualitative instrument is influential. The client started with general areas to specific topics. In her initial FCA, this client stated, “Some possibilities of what I might want to do are either a writer or a musician.” In her later FCA, she stated, “I want to write a book after I have a stable job.” Her FCA shifted from an initial sense of vagueness about her career to a narrative with a clear direction. The client had accepted the need to move on and the FCA moved from fixation to an openness theme. For changes in narrative themes, the case's FCA changed substantially in the content after counseling, suggesting that the counseling changed the case's narrative ability.

Reconceptualization of innovative moments is associated with psychotherapeutic gains. Reconceptualizing IMs could improve symptoms of depression (Fernández-Navarro et al., 2018). The emergence of reconceptualized IMs indicates that the intervention was effective. The case gradually became aware that the biggest problem was her unwillingness to learn mathematics. The career problem was transformed into a real problem, and the awareness was translated into action. As a result of the life design counseling, the client became more attentive and brave to ask questions in math class. The client realizes that only she can decide her career and have a greater sense of control over her career. The client moved from disinterest and uncertainty to actively look for career information, and finally, specific action goals and solutions are set in the plan.

## 8. Recommendations for future research

The challenges of the LDC approach include the lack of focus on clients from multiple backgrounds and professional counselors and the lack of diversified methods and research in the intervention process (Wen et al., 2022). The findings of our study may have important implications for the evaluation of life design intervention and career counseling. However, since only one case was investigated in this study, this limits the possibility of applying these results to other cases.

Future research will expand into this type of group to refine a more targeted model of career counseling. It should also develop different forms of creative career construction interviews, such as written exercises, career collages, and career portfolios, to accommodate students of different ages. In addition, future research should focus on the sustainability of an individual's career adaptability and sense of self after participating in a life design intervention program. And finally, it would be possible to link professional and psychological behaviors and to integrate life design counseling and psychotherapy practice.

## 9. Conclusion

This study aimed to establish the influence of LDC on a high school student's indecision in choosing the subject. Based on the findings, the case study based on LDC methods can be regarded as an effective process intervention for high school students facing choice problems. In addition, the case study confirmed the effectiveness of the evaluation (process evaluation and results' evaluation). In summary, LDC is suitable for high school students who are hesitant before choosing a subject, and the approach can improve their career adaptability and promote career exploration.

## Data availability statement

The raw data supporting the conclusions of this article will be made available by the authors, without undue reservation.

## Ethics statement

Ethical review and approval was not required for the study on human participants in accordance with the local legislation and institutional requirements. Written informed consent to participate in this study was provided by the participants' legal guardian/next of kin.

## Author contributions

DW and XL contributed to design of the study. DW wrote the manuscript. All authors contributed to the article and approved the submitted version.
